# P-549. Safety and Efficacy of Baloxavir Marboxil for Influenza Treatment in Chinese Children Aged 1-<5 Years: A Single-Arm Multicenter Clinical Trial

**DOI:** 10.1093/ofid/ofaf695.764

**Published:** 2026-01-11

**Authors:** Yuchuan Li, Peng Guo, Chenguang Jia, Ju Yin, Yuncui Yu, Qiang Qin, Xiaoyan Zhang, Shunying Zhao, Haiming Yang, Weihua Zhang, Yuguang Liang, Qian Ding, Man Tian, Yiping Chen, Chunmei Zhu, Fang Wang, Jing Ma, Lisu Huang, Yongping Xie, Jing Bi, Sainan Shu, Chengsong Zhao

**Affiliations:** Beijing Children's Hospital, Capital Medical University, National Center for Children's Health, Beijing, Beijing, China; Beijing Children's Hospital, Capital Medical University, National Center for Children's Health, Beijing, Beijing, China; 1.Beijing Children's Hospital, Capital Medical University, National Center for Children's Health;2.Shunyi Maternity and Child Hospital, Beijing Children's Hospital, Capital Medical University, Beijing, Beijing, China; Beijing Children's Hospital, Capital Medical University, National Center for Children's Health, Beijing, Beijing, China; Beijing Children's Hospital, Capital Medical University, National Center for Children's Health, Beijing, Beijing, China; Beijing Children's Hospital, Capital Medical University, National Center for Children's Health, Beijing, Beijing, China; Beijing Children's Hospital, Capital Medical University, National Center for Children's Health, Beijing, Beijing, China; Beijing Children's Hospital, Capital Medical University, National Center for Children's Health, Beijing, Beijing, China; Beijing Children's Hospital, Capital Medical University, National Center for Children's Health, Beijing, Beijing, China; Beijing Children's Hospital, Capital Medical University, National Center for Children's Health, Beijing, Beijing, China; Beijing Children's Hospital, Capital Medical University, National Center for Children's Health, Beijing, Beijing, China; Beijing Children's Hospital, Capital Medical University, National Center for Children's Health, Beijing, Beijing, China; Nanjing Children's Hospital, Nanjing, Jiangsu, China; The Second Affiliated Hospital and Yuying Children's Hospital of Wenzhou Medical University, Wenzhou, Zhejiang, China; Children's Hospital, Capital Institute of Pediatrics, Beijing, Beijing, China; Children's Hospital Affiliated to Zhengzhou University, Henan Children's Hospital, Zhengzhou Children's Hospital, Zhengzhou, Henan, China; Jinan Children’s Hospital, Jinan, Shandong, China; Children's Hospital Zhejiang University School of Medicine, Hangzhou, Zhejiang, China; Children's Hospital Zhejiang University School of Medicine, Hangzhou, Zhejiang, China; Baoding Hospital, Beijing Children's Hospital affiliated to Capital Medical University, Baoding, Hebei, China; Tongji Hospital, Tongji Medical College of Huazhong University of Science and Technology, Wuhan, Hubei, China; Beijing Children's Hospital, Capital Medical University, National Center for Children's Health, Beijing, Beijing, China

## Abstract

**Background:**

Influenza causes significant morbidity in children under 5, with high rates of hospitalization and complications. Baloxavir marboxil offers clinical benefits through its single-dose regimen and rapid antiviral activity in influenza treatment. While currently approved for individuals 5 years of age and older in China, the safety and efficacy data in children (1-< 5 years) are limited. This study presents the primary analysis evaluating the safety and efficacy of baloxavir marboxil in Chinese children aged 1 to < 5 years with influenza.

**Methods:**

We conducted a single-arm, multicenter study in Chinese children aged 1 to < 5 years with laboratory-confirmed influenza A and/or B (symptom onset ≤48 hours). Eligible participants received a single oral dose of baloxavir marboxil suspension based on body weight (2 mg/kg for < 20 kg; 40 mg for ≥ 20 kg to < 80 kg). The primary endpoint assessed the incidence and severity of adverse events, including serious adverse events. Secondary endpoints evaluated the time to alleviation of influenza signs and symptoms (TTASS), fever duration, incidence of influenza-related complications, and virus detection in serial respiratory swabs.

**Results:**

During the 2024-2025 influenza season, we enrolled 100 influenza patients. Overall, 34 adverse events (AEs) were reported in 29 (29%, 29/100) children, with SAEs occurring in 3% (3/100) of participants. The most common AEs included bronchitis (4%), diarrhea (4%), upper respiratory tract infection (3%) and cough (3%). Only one case (rash) was determined by the investigators to be drug-related AE, which was grade 1. The median TTASS was 150.17 hours (95% CI, 90.23-NE), with fever resolution occurring within a median of 38.65 hours (95% CI, 25.32-42.35). The median time to cessation of viral shedding by virus titer was 22.74 hours (95% CI, 22.20-24.58). Influenza-related complications developed in 5% of participants, while systemic antibiotics were administered in none of the cases.

**Conclusion:**

In this multicenter evaluation, weight-adjusted baloxavir marboxil demonstrated a favorable safety profile and clinically meaningful efficacy for the treatment of influenza in Chinese children aged 1 to < 5 years.

**Disclosures:**

Chengsong Zhao, MD, Roche: Grant/Research Support

